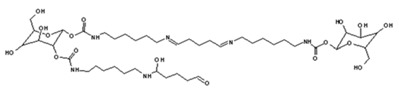# Preparation and Evaluation of Glucose Based Non-Isocyanate Polyurethane Self-Blowing Rigid Foams

**DOI:** 10.3390/polym11111802

**Published:** 2019-11-02

**Authors:** Xuedong Xi, Antonio Pizzi, Christine Gerardin, Hong Lei, Xinyi Chen, Siham Amirou

**Affiliations:** 1 LERMAB, University of Lorraine, 27 rue Philippe Seguin, 88000 Epinal, France; xuedong.xi@univ-lorraine.fr (X.X.); xinyi.chen@univ-lorraine.fr (X.C.); siham.amirou@univ-lorraine.fr (S.A.); 2 Yunnan Key Laboratory of Wood Adhesives and Glue Products, Southwest Forestry University, Kunming 650224, China; honeyray2006@hotmail.com; 3 Department of Physics, King Abdulaziz University, Jeddah 21589, Saudi Arabia; 4 LERMAB, University of Lorraine, Boulevard des Aiguillettes, 54000 Nancy, France; christine.gerardin@univ-lorraine.fr

**Keywords:** non-isocyanate polyurethane, NIPU, glucose, rigid foams, self-blowing

## Abstract

A partially biobased self-blowing and self-hardening polyurethane foam from glucose-based non-isocyanate polyurethanes (g-NIPU) was prepared by reaction of glucose with dimethyl carbonate and hexamethylene diamine. However, these foam types generally require a high foaming temperature. In this paper, a self-blowing foam based on g-NIPU was prepared at room temperature by using maleic acid as an initiator and glutaraldehyde as a crosslinker. Water absorption, compression resistance, and fire resistance were tested. Scanning electron microscopy (SEM) was used to observe the foam cells structure. Middle infrared (ATR FT-MIR) and Matrix Assisted Laser Desorption Ionization Time-of-Flight (MALDI-TOF) mass spectrometry were used to help to analyze the reactions during the foaming process. The results obtained showed that self- blowing rigid foams have good compression, this being directly proportional to the foam density. Increasing the amount of glutaraldehyde or reducing maleic acid thickens the cell walls and increases the density of the foams. MALDI-TOF analysis showed that g-NIPU reacts with both maleic acid and glutaraldehyde. The foams presented poor fire resistance indicating that, as for isocyanate based polyurethane foams, addition of a fire retardant would be necessary.

## 1. Introduction

As heat insulation foams are widely used in daily life and industry, where light weight, good sound absorption and shock absorption material characteristics are sought after. Today polyurethane (PU) foams have a wide range of uses in the decoration, construction, packaging, refrigeration, automotive and other industries due to their mature technology and high output. The traditional synthesis route of PU is based on the reaction of polyols with polyisocyanates. However, due to polyisocyanates (such as toluene diisocyanate (TDI), hexamethylene diisocyanate (HDI)) having strong volatility, toxicity, and being harmful to the environment, non-isocyanate polyurethanes (NIPU) have been synthesized [1,2], to eliminate these drawbacks of isocyanate-based polyurethanes. 

**Scheme 1:**
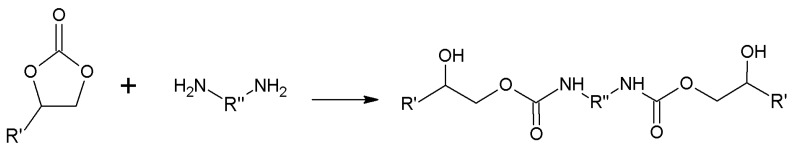
The main synthetic route to non-isocyanate polyurethane (NIPU).

Currently, the main route to the preparation of NIPU is by reacting a cyclic carbonate with a primary amine (Scheme 1).

In general, there are two very common methods for preparing cyclic carbonates. By the reaction of an epoxy group with CO_2_, under catalyst and pressure conditions, which has been widely reported in the literature [3,4,5,6,7,8,9,10,11]. It is on this approach that the more interesting research on the preparation of biomass-based NIPU by biomass materials such as soybean oil and rapeseed oil have been already presented. Such a kind of biobased NIPU has been used for coating, plastics and rigid foams [12,13,14,15,16,17]. 

A second effective method is by reacting dimethyl carbonate with o-hydroxyl chemicals to obtain cyclic carbonates [18], followed by reaction with a diamine [9,10,19,20,21,22,23]. As dimethyl carbonate is a non-toxic and an environmentally friendly chemical, and moreover as the reaction occurs under easy conditions this approach makes this preparation method highly valued. However, an even easier approach to the preparation of NIPUs is based on the reaction of bio-materials such as tannins [24,25,26], lignin [27], and sugars [28,29] with just dimethyl carbonate, thus without the need of cyclization of the carbonate, and a diamine. This approach has been recently reported, and the NIPUs so prepared were used as wood coatings and wood bonding adhesives [28,29]. 

More recently, the preparation of a bio-based NIPU foam from glucose with dimethyl carbonate and diamine has been reported [30]. This was an open cell foam with good properties, particularly in that compression can only flatten the cell walls, with the cellular structure being maintained and the cell walls not been destroyed, thus showing recuperation of form. However, its severe drawback is that it requires a high temperature for foaming (220 °C), this being a real constraint for certain applications. Thus, there is an interest in developing a way of foaming these materials at a lower temperature. In the work presented here, a self-blowing foam from a glucose-based NIPU was prepared by foaming at room temperature and hardening for a limited time at a much lower temperature (103 °C) than before, with maleic acid as an initiator, followed by analyzing its properties.

## 2. Materials and Methods

### 2.1. Materials

The glutaraldehyde (C_5_H_8_O_2_, 50% water solution) used was of analytical grade (AR), obtained from Acros Organics (Illkirchn France); Glucose by Acros organics (Geel, Belgium), dimethyl carbonate (AR) by Sigma-Aldrich (Saint-Quentin Fallavier, France); hexamethylenediamine (AR) by Sigma-Aldrich (Saint-Quentin Fallavier, France), maleic acid (AR) by Sigma-Aldrich (Saint-Quentin Fallavier, France), the glutaraldehyde by Sigma-Aldrich (Saint-Quentin Fallavier, France). The 2,5-dihydroxybenzoic acid (DHB) by Acros Organics (Illkirch, France).

### 2.2. Synthesis of the Glucose Based Non-Isocyanate Polyurethane(g-NIPU)

The glucose NIPUs were prepared according to a procedure already reported [28]: 160 g of glucose was mixed with 106.7 g of dimethyl carbonate and 133.36 g of water in a three-necked flask with reflux condenser, and heated to 50 °C for 40 min. Then 310.4 g hexamethylenediamine (70% solution) was added to the mixture, heated to 90 °C for 60 min, then cooled to room temperature.

### 2.3. Preparation of NIPU Foams

The glucose-NIPU was mixed with maleic acid and glutaraldehyde for foaming at room temperature. The self-sustaining soft foam so obtained was maintained at ambient temperature (25 °C) for 5 h, then it was dried in an oven at 103 °C for 4 h. The foam was then removed from the foaming beaker and placed at ambient temperature (25 °C and 12% relative humidity) for 2 days before its characterization. The foams prepared were tested for their water absorption by keeping them immersed in water at ambient temperature for two hours. The composition and mass ratio of the mixture are shown in Table 1.

Compared to the method in the literature that showed the preparation of NIPU foams at 220 °C [30], the foaming process in this work was carried out at room temperature, and without a blowing agent being used. It is an easier method and also reduces energy consumption. 

### 2.4. FTIR

To confirm the presence of the relevant structures, the samples extracts were analyzed with a Perkin-Elmer Frontier ATR-FT-MIR provided with an ATR Miracle diamond crystal. The powder and liquid samples were laid on the diamond eye (1.8 mm) of the ATR equipment and the contact for the sample was ensured by tightly screwing the clamp device. Each extract was scanned registering the spectrum with 32 scans with a resolution of 4 cm^−1^ in the wave number range between 600 and 4000 cm^−1^.

### 2.5. MALDI-TOF Analysis

Samples for Matrix Assisted Laser Desorption Ionization Time-of-Flight (MALDI-TOF) mass spectrometry analysis were prepared by first dissolving 5 mg of sample powder in 1 mL of a 50:50 *v*/*v* acetone/water solution. Then 10 mg of this solution was added to 10 µL of a 2,5-dihydroxy benzoic acid (DHB) matrix. The locations dedicated to the samples on the analysis plaque were first covered with 2 µL of a NaCl solution 0.1 M in 2:1 *v*/*v* methanol/water, and pre-dried. Then 1 µL of the sample solution was placed on its dedicated location and the plaque was dried again. MALDI-TOF spectra were obtained using an Axima-Performance mass spectrometer from Shimadzu Biotech (Kratos Analytical Shimadzu Europe Ltd., Manchester, UK) using a linear polarity-positive tuning mode. The measurements were carried out making 1000 profiles per sample with 2 shots accumulated per profile. The spectrum precision is of +1 Da.

### 2.6. Water Absorption (24 h)

In order to investigate the water absorption of glucose-NIPU foams and the effect of their densities on water absorption, 4 samples (2 cm × 2 cm × 2 cm) were tested for their 24 h water absorption.

### 2.7. Compression

Samples of 2 cm × 2 cm × 2 cm were cut from foams obtained by different formulations and were compression tested. The tests were done under compression on an Instron 3300 dual column universal testing machine (Instron France, Elancourt, France) at a head rate of 1 mm/min.

### 2.8. Ignition Test

**Figure 1:**
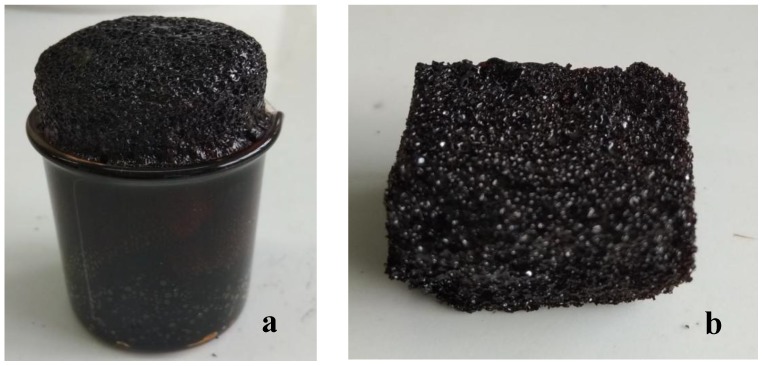
Glucose based non-isocyanate polyurethane foams (“**a**” in the mold, “**b**” after cutting it).

According to literature methods [30], foam samples of 2.5 cm × 2.5 cm × 2.5 cm (Figure 1b) cut from the foams prepared (Figure 1a) were placed in a porcelain crucible preheated on a Bunsen burner and with the crucible at 600 °C. Heating with the burner is maintained at the same temperature until the sample chars and does not burn anymore and the time to reach this state is measured. The test is done in duplicate for each type of foam to be tested.

## 3. Results and Discussion

### 3.1. Effect of Amount of Maleic Acid Addition on Performance of the Foams

Some basic properties such as density, 2 h water absorption and fire resistance of the foams were tested, and the results are shown in Table 2. From the formulations for preparing the NIPU foams in Table 1, when increasing maleic acid addition (F2, F3, F4 in Table 1 and Table 2), the density of the foams decreased. Due to the rather lively reaction of maleic acid with hexamethylene diamine, more energy was produced thereby generating more foamed material or larger foam cells. This can be observed by scanning electron microscopy (SEM). Consequently, because of this, as the amount of acid increases the density of the foams also decreases. As already reported these self-blowing g-NIPU foams have also poor fire resistance [30]. Although slightly better in this respect than previously reported, these foams are clearly not fire resistant, and as for isocyanate based polyurethane foams, fire retardants would need to be added. In standard commercial isocyanate-based polyurethane foams, fire retardants are regularly added to both slow down burning as well as to render the foam self-extinguishing to limit the possibility of fire and of melting of the foam if alight. The new foams based on glucose NIPU presented here are no exception to this need, with the requirement for fire retardant addition to the same extent and for the same purpose. In addition, it can be observed that the fire resistance is related to foam density, as the ignition time shortened as the density is reduced. This seems to imply a reduction in the fire resistance of the foam, but in reality it is simply due to a reduction in the combustible material, which constitutes the cell wall, thus resulting in a reduction in the combustion time of the same volume of foam. Larger density foams also show a greater 2 h water absorption, which is logical, as water absorption is caused by the glucose of the g-NIPU that makes up the cell wall, and higher density foams have thicker cell walls, as observed in the SEM test results.

### 3.2. Effect of Glutaraldehyde Addition Amounts on Basic Performance of Foams

Foams prepared with different amounts of glutaraldehyde were obtained and tested and some of their characteristics are shown in Table 2. By comparing foams F5 and F6, it appears that increasing the proportion of glutaraldehyde addition can result in an increase in foam density, while also causing a higher 2h water absorption and a longer ignition time. The most likely explanation for this is that the cell walls of the foam were thicker. The reaction of glutaraldehyde with the –NH– groups in g-NIPU [31,32] causes the viscosity of the mixture to sharply increase and then gel during the foaming process, which results in expansion or foaming being more difficult. This means that mixtures of the same quality can only expand to a smaller volume, thus having a greater density and thicker cell walls.

**Figure 2:**
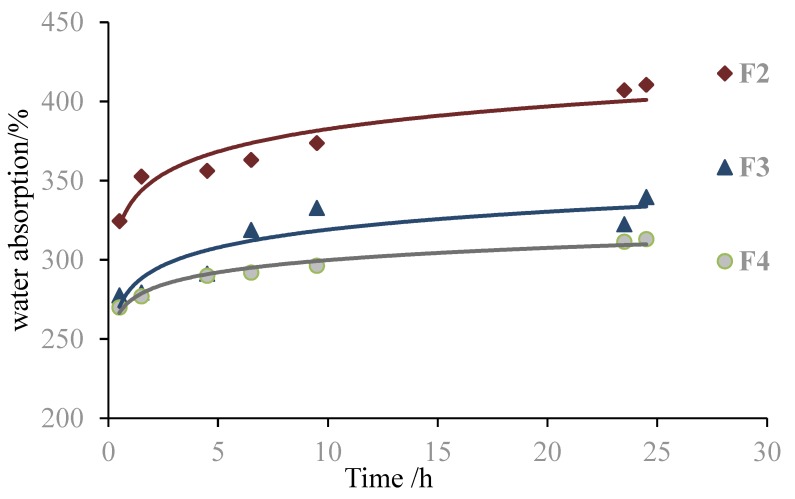
Water absorption (24 h) of g-NIPU foams with different maleic acid (MA) addition: F2 (10g g-NIPU + 1 g MA + 2.5 g G), F3 (10 g g-NIPU + 1.2 g MA + 2.5 g G), F4 (10 g g-NIPU + 1.5 g MA + 2.5 g G).

**Figure 3:**
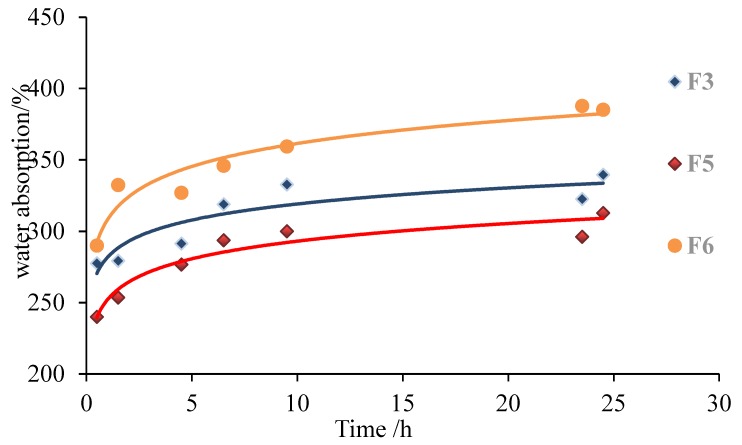
Water absorption (24 h) of g-NIPU foams with different glutaraldehyde addition: F3 (10 g g-NIPU + 1.2 g MA + 2.5 g G), F5 (10 g g-NIPU + 1.2 g MA + 1.8 g G), F6 (10 g g-NIPU + 1.2 g MA + 3.6 g G).

Figure 2 and Figure 3 show the 24 h water absorption of g-NIPU foams prepared as for the formulations in Table 1. First, foams in both Figure 2 and Figure 3 exhibit different levels of water absorption, this being directly related to their densities. Furthermore, the water absorption levels markedly increase during the first five test hours, due to the storage of water in the cell cavity. The water absorption levels off after about 5 h water immersion and appears to be in direct relation to foam density, as the curves for lower density foams (F3, F4) tend to be steady, while those of the denser foams (F2, F6) continue to increase, albeit at a slower rate.

### 3.3. Foam Compression Resistance

**Figure 4:**
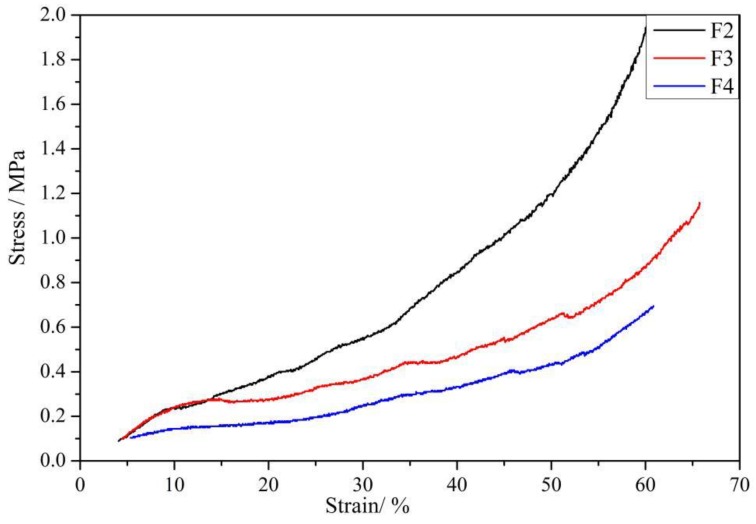
Effect of different amounts of maleic acid (MA) addition on compression.

**Figure 5:**
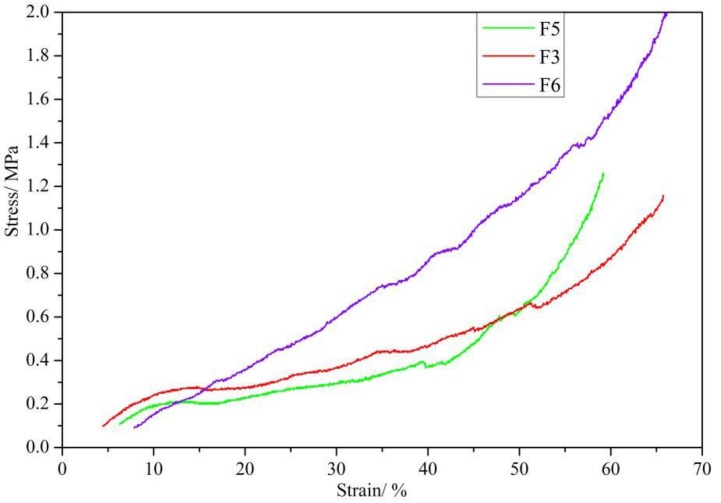
Effect of different amounts of glutaraldehyde addition on compression.

Figure 4 shows the stress–strain curves in compression for foams F2, F3, and F4 prepared with different amounts of maleic acid (Table 1). The mechanical resistance of foam F2 is better than the others, due to its much higher density, while the minimum density foam F4 presents the weakest stress–strain curve. It is known that compression resistance is directly proportional to foam density [30,33], as the amount of acid addition increases, the density of the foams decreases and thus it presents a lower compression resistance. The stress–strain curves in compression of foams F3, F5, and F6 prepared with different amounts of glutaraldehyde additions are presented in Figure 5. The F6 foam has the highest stress–strain curve because of its maximum density, which is the same as the F2 foam in Figure 4. The stress–strain curves of F3 and F5 are similar to each other due to their densities being very similar. Thus, the addition of acid and glutaraldehyde has a direct effect on the density of the foams, which in turn determines their compressive properties.

In brief, the addition of maleic acid and glutaraldehyde affects the compression properties of the foams. Increasing the amount of maleic acid or reducing the amount of glutaraldehyde will reduce the compression resistance of the foams, as both of them change the density of the foam. Nevertheless, the foams obtained in this work still present good compression properties, at the same level as described in the literature [30].

### 3.4. SEM Analysis

**Figure 6:**
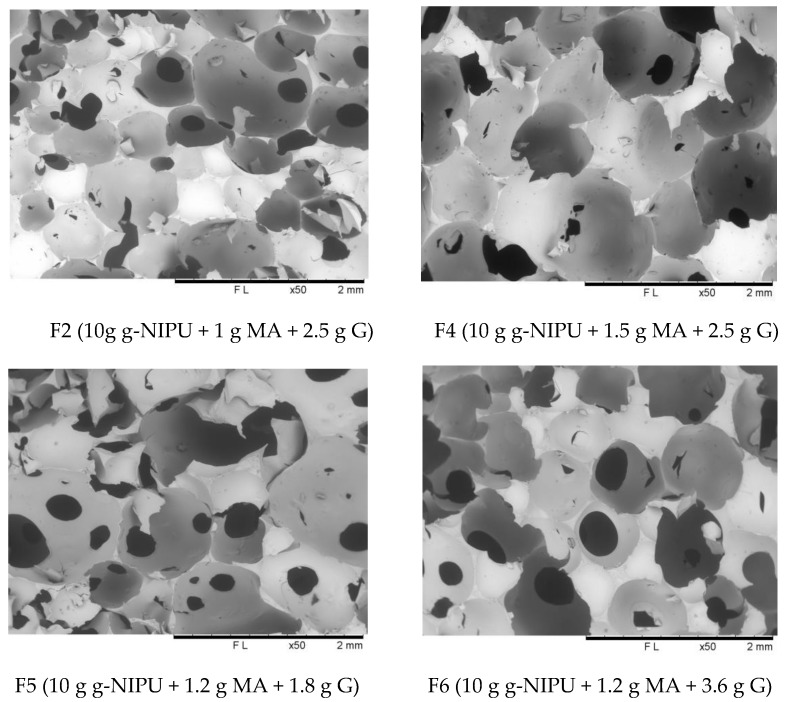
Scanning electron microscopy (SEM) pictures of self-blowing foams.

To interpret the effect of different amounts of glutaraldehyde/maleic acid on foam cell structures, scanning electron microscopy (SEM) pictures of foams F2, F4, F5, F6 were observed and are shown in Figure 6. First, it is easy to see that there are two different types of cells in the foams, namely open and closed cells. Second, comparing pictures of F2 and F4 in Figure 6, it can be seen that foam F2 has a thicker cell wall, explaining its higher density in Table 2 and the better compression resistance in Figure 4. In addition, foam F4 appears to have larger cells than F2, also giving the fact that F4 has a greater porosity and a smaller density. 

Figure 6 shows that foam F6, prepared with a larger amount of glutaraldehyde addition has thicker cell walls and smaller cells. This again demonstrates and supports the fact that the rapid reaction between glutaraldehyde and g-NIPU causes the foaming system to gel and the foamed mixture to be harder. 

In general, an increase in the amount of maleic acid in this system leads to enlarged and thinner foam cells. However, increasing the proportion of glutaraldehyde gives a foam with the exact opposite result, that is, smaller cells and thicker cell walls. 

### 3.5. FTIR Analysis

**Figure 7:**
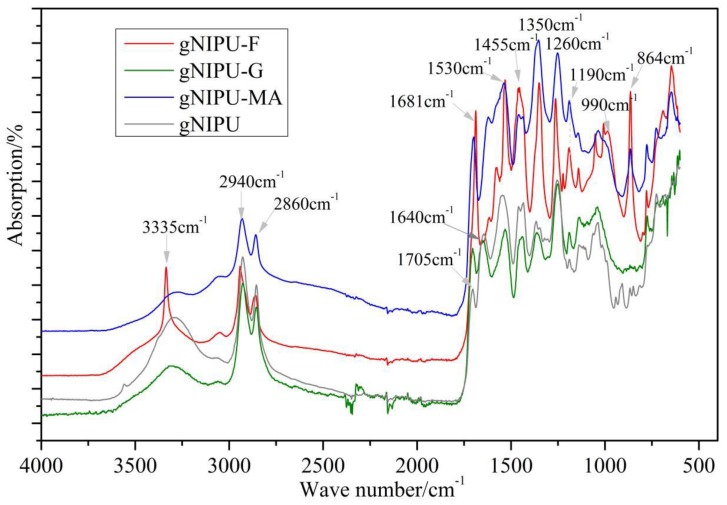
FTIR spectra of g-NIPU, g-NIPU-G, g-NIPU-MA and g-NIPU foams (g-NIPU-F).

The FTIR spectra of the glucose non-isocyanate polyurethanes (g-NIPU), the reaction product of the g-NIPU with maleic acid (g-NIPU-MA), the reaction product of the g-NIPU with glutaraldehyde (g-NIPU-G), and the foams prepared in this work(g-NIPU-F) are shown in Figure 7. 

The wide peak around 3300 cm^−1^ is characteristic of the O–H and N–H groups [34], which is decreased in g-NIPU-MA and g-NIPU-G when compared with g-NIPU, this being so because maleic anhydride or glutaraldehyde react with the amine groups in g-NIPU to consume it. The sharp bands at 2860 and 2940 cm^−1^ are attributed to the –CH_2_– stretching vibration [35]. It is very clearly visible in the curves of g-NIPU and g-NIPU-G with signals appearing at 1700 and 1640 cm^−1^, which are typical for carbonyl IR absorption of urethane groups and N–H deformation of the urethane group, respectively [4,34]. The peak at 1640 cm^−1^ has disappeared with the presence of maleic acid in the system of g-NIPU-MA and the foams g-NIPU-F, because of the reaction between the carboxyl and the amino groups to form amide bonds. Meanwhile, due to the hydrogen of the N–H bond in the urethane groups being replaced, the C=O vibration peak at 1700 cm^-1^ shifts to a lower wavenumber of 1681 cm^−1^ [34,36]. The absorption at peak 990 cm^−1^ in the curves of g-NIPU-MA and g-NIPU-F is attributed to the C=C linkages [34], which are present once maleic acid has been added. A sharp absorption peak at 3335cm^−1^ indicated the presence of N–H stretching vibration from amide bonds, which is possibly the result of the reaction of maleic acid with free hexamethylene diamine or amine groups in g-NIPU; this is confirmed by the structure analysis by MALDI-ToF which follows. 

### 3.6. MALDI ToF Analysis

**Figure 8:**
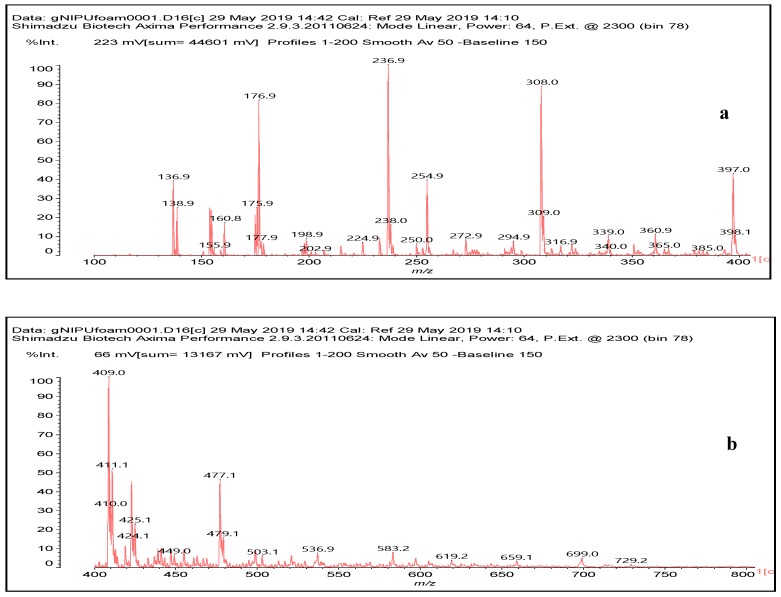

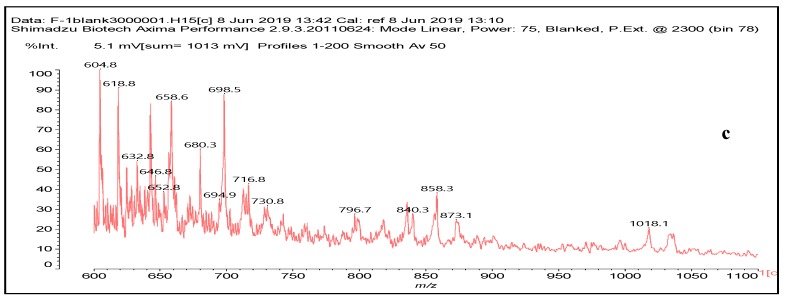
MALDI ToF spectrum of the self-blowing foam: (**a)** 100–400 Da range, (**b)** 400–800 Da range, (**c)** 600–1100 Da range.

In order to better analyze the chemical structures of the self-blowing foams prepared and describe the progress of the reaction during foaming, a hardened foam F3 was analyzed by MALDI ToF. The results are shown in Figure 8, and their interpretation in Table 3. First, it must be considered that the molecular weight of the hardened foams obtained is so high as to be out of scale for MALDI ToF analysis. Thus, only some smaller molecular weight species can be identified, but this is already enough to help to understand the reactions occurring during foaming. Maleic acid is used as an initiator, which reacts with the excess of free hexamethylene diamine in the NIPU mixture to provide foaming power as shown by the species at 237 Da, namely:


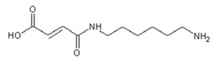


and 410 Da of structure as:


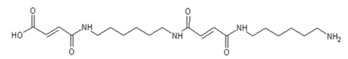


This is confirmed by the results of the FTIR analysis [34,36]. These species can also further react with NIPU, as shown by the peak at 541 Da:


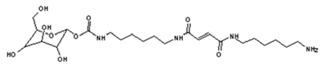


Glutaraldehyde can be considered as a cross-linking agent in the system, reacting with the amino groups of NIPU to link two NIPU molecules, as shown by the species at 731 Da:


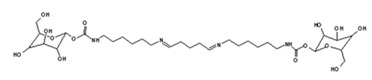


The same is valid for the peak at 873 Da, because of the easy reaction between the amine group and the aldehyde [37,38]:


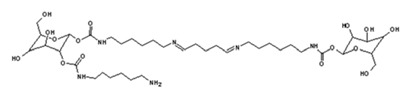


More evidence suggests also that maleic acid and glutaraldehyde can simultaneously react with g-NIPU, such as shown by the species at 858 Da, as:


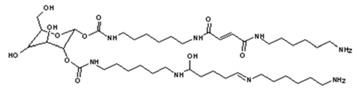


All of these branched molecules provide the possibility of forming a three-dimensional network, and to ensure that the foam after hardening has a good performance. Due the problem of water sensitivity of imines [39,40], it is worth noting that the imine bond(C=N) in the molecular structures reacts easily with water in the rearrangement from a C=C bond to a –COH–N– group, it is also a cause of water absorption of the foams.

## 4. Conclusions

Glucose-biobased non-isocyanate polyurethane (g-NIPU) rigid foam has already been reported [32], by reaction with NaHCO_3_ as a blowing agent and addition of a silane coupling agent as a crosslinker. While these foams are of interest to avoid the use of an isocyanate they also present a drawback in that they are foamed and hardened by applying heat at a relatively high temperature. In this paper a room temperature, self-blowing g-NIPU foam preparation method is presented and implemented eliminating the need for high temperature foaming for these g-NIPUs and this without a blowing agent being used [30]. These foams can be used as thermal insulation materials, and/or as a middle layer to make a lightweight "wood–foam–wood" sandwich board, or even for hydroponic applications [41]. Both open and closed cells can be observed in these foams, and they also present good resistance to compression, but still present a poor fire resistance. More research on the fire resistance modification of these foams is needed. Maleic acid as initiator and glutaraldehyde as a crosslinker are used for the foaming reaction. Increasing the amount of glutaraldehyde or reducing the maleic acid thickens the cell walls and increases the density of the foams, thus affecting their compression performance. 

## Figures and Tables

**Table 1:** Formulations for preparing the non-isocyanate polyurethane (NIPU) foams.

No.	g-NIPU (g)	Maleic Acid (g)	Glutaraldehyde (g)
F2	10	1.0	2.5
F3	10	1.2	2.5
F4	10	1.5	2.5
F5	10	1.2	1.8
F6	10	1.2	3.6

**Table 2:** Performance of the g-NIPU foams.

No.	Formulations	Density (g/cm3)	2 h Water Absorption (%)	Ignition Time (s)
F2	10 g g-NIPU + 1.0 g MA + 2.5 g G	0.13	352.6	140
F3	10 g g-NIPU + 1.2 g MA + 2.5 g G	0.09	279.3	120
F4	10 g g-NIPU + 1.5 g MA + 2.5 g G	0.08	276.9	88
F5	10 g g-NIPU + 1.2 g MA + 1.8 g G	0.10	253.5	130
F6	10 g g-NIPU + 1.2 g MA + 3.6 g G	0.13	332.4	175

**Table 3:** Proposed oligomer structures for the MALDI ToF spectra peaks in Figure 7 for the self-blowing foam.

236.9 Da (calc. 237) = Small molecule of maleic acid reacts with hexamethylenediamine with 23 (Na+)
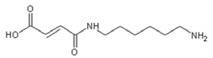
322 Da (calc. 322) = molecule of urethane structure of “glucose–carbonate–diamine” without Na+
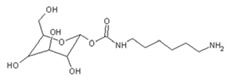
409–411 Da (calc. 410) = reaction results of two molecules “maleic acid–diamine” without Na+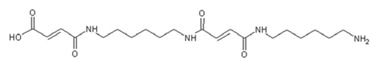
422 Da = 322Da react with glutaraldehyde without Na+
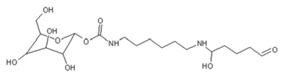
520 Da = 422 Da react with diamine without Na+
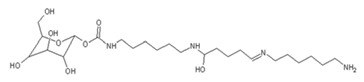
528 Da = urethane structure of “glucose–carbonate–diamine–carbonate–glucose” without Na+
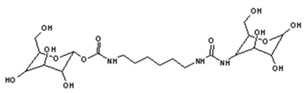
541 Da = molecule of “glucose–carbonate–diamine–maleic acid–diamine” with Na+
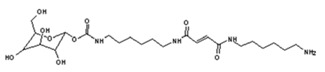
564 Da = urethane structure of “glucose–carbonate–diamine–carbonate–diamine” with one aldehyde without Na+
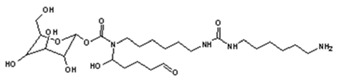
618 Da = molecule of “glucose–carbonate–diamine–aldehyde–diamine–maleic acid” without Na+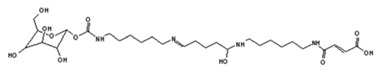
644 Da = molecule of “glucose–2×carbonate–2×diamine–aldehyde–maleic acid” without Na+
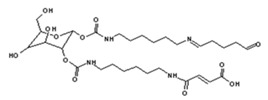
731 Da = molecule of” 2×glucose–carbonate–diamine” connected with aldehyde as a bridge, with Na+
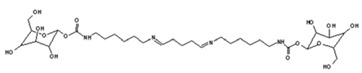
858 Da = Dendrimer of “glucose–2×carbonate–4×diamine–aldehyde–maleic acid” without Na+
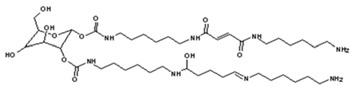
873 Da = 731 Da react with carbonate and diamine, without Na+
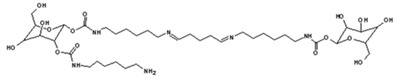
1019 Da = 873 Da react with one aldehyde, with Na+